# Robotic Distal Gastrectomy with D2 Lymphadenectomy for Gastric Cancer in a Patient with an Unclassifiable Celiac Arterial Anomaly: A Case Report

**DOI:** 10.70352/scrj.cr.26-0317

**Published:** 2026-09-10

**Authors:** Kohei Fujita, Hiroyuki Sagawa, Shigeyuki Kosaka, Shohei Hayashi, Eri Tsuji, Shunsuke Hayakawa, Sunao Ito, Reo Sato, Ryo Ogawa, Shuji Takiguchi

**Affiliations:** Department of Gastroenterological Surgery, Nagoya City University Graduate School of Medical Sciences and Medical School, Nagoya, Aichi, Japan

**Keywords:** gastric cancer, robotic surgery, vascular anomaly

## Abstract

**INTRODUCTION:**

Suprapancreatic lymph node dissection during gastric cancer surgery is technically challenging, particularly in the presence of celiac arterial system–related vascular anomalies. However, some rare arterial configurations do not clearly fit within existing classification systems, making surgical planning difficult. Herein, we report a case of gastric cancer with an unclassifiable celiac arterial anomaly that was managed using a robotic approach.

**CASE PRESENTATION:**

A 77-year-old woman presented with several weeks of persistent epigastric pain and was referred to our hospital. Imaging studies revealed gastric cancer and an unusual vascular anatomy in which the left gastric, splenic, common hepatic, and superior mesenteric arteries arose independently from the abdominal aorta, with the common hepatic artery coursing dorsal to the portal vein. The patient underwent robotic distal gastrectomy with D2 lymphadenectomy. Preoperative 3D-CTA enabled accurate anatomical assessment. Portal vein taping facilitated safe suprapancreatic lymph node dissection. The postoperative course was uneventful.

**CONCLUSIONS:**

This case demonstrates that even unclassifiable vascular anomalies can be safely managed with detailed preoperative anatomical assessment and appropriate intraoperative strategies, providing a practical approach for surgical decision-making in anatomically complex gastric cancer cases.

## Abbreviations

CA19-9
: carbohydrate antigen 19-9
CEA
: carcinoembryonic antigen
CHA
: common hepatic artery
LGA
: left gastric artery
SA
: splenic artery
SMA
: superior mesenteric artery
UICC
: Union for International Cancer Control

## INTRODUCTION

Suprapancreatic lymph node dissection during gastric cancer surgery is technically demanding as major vessels, including the CHA, portal vein, and SMA, are densely clustered within a confined anatomical space.^1)^ In patients with celiac arterial anomalies, the lymphadenectomy-related complexity increases substantially, with a reportedly increased risk of intraoperative bleeding and vascular injury.^2,3)^ Accurate preoperative vascular anatomy assessment and individualized surgical planning are thus essential for patient safety.

Several classification systems, including the Adachi classification, have been proposed to categorize celiac and superior mesenteric arterial system–related variations and have contributed to anatomical understanding and surgical risk assessment.^4,5)^ However, these systems are based on representative branching and course patterns. Consequently, certain atypical cases with combined anomalies of arterial origin and spatial course do not strictly fit within existing frameworks.

Although unclassifiable vascular anomalies remain uncommon, they could significantly affect suprapancreatic lymph node dissection. The CHA is a key landmark in this region, and deviations from its usual course might induce hemorrhage, pancreatic injury, or inadequate lymphadenectomy.^2,3)^

Robotic gastrectomy is increasingly used in anatomically complex cases because it offers high-resolution 3D visualization and articulated instruments for precise dissection.^6,7)^ However, these advantages depend on meticulous preoperative anatomical assessment and appropriate intraoperative exposure.

Herein, we report a case of a rare and unclassifiable vascular anomaly-associated gastric cancer, with the LGA, SA, CHA, and the SMA all arising independently from the abdominal aorta, and the CHA coursing dorsal to the portal vein. Detailed preoperative CT allowed for implementing a planned surgical strategy incorporating portal vein taping, thereby yielding safe and reliable suprapancreatic lymph node dissection during robotic surgery.

## CASE PRESENTATION

**Fig. 1:**
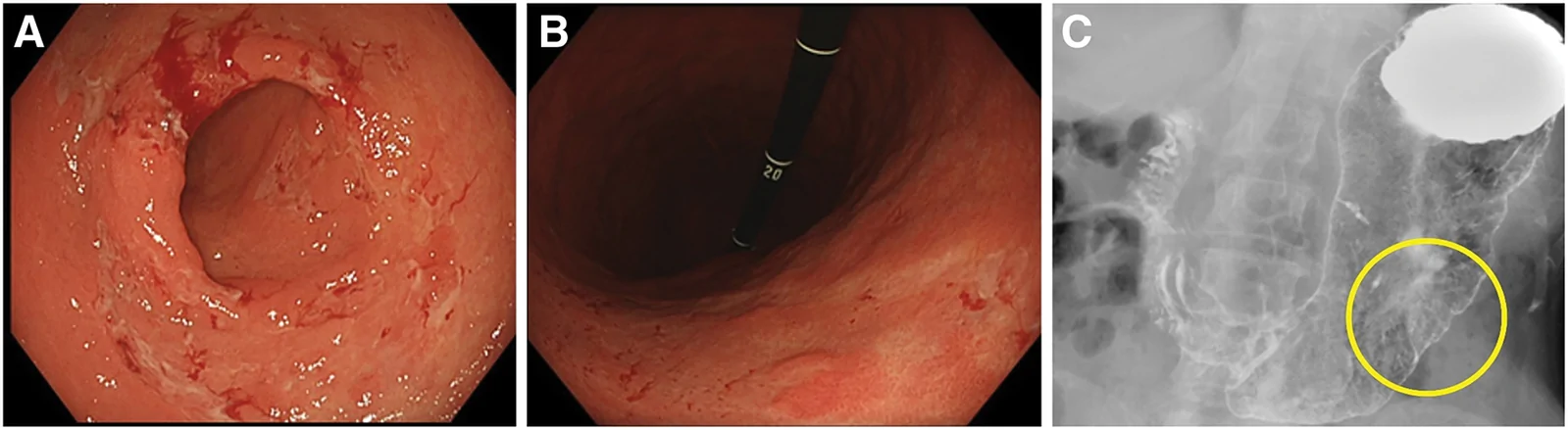
Endoscopic and radiographic findings of gastric cancer. (**A**, **B**) Upper gastrointestinal endoscopy showing an irregular ulcerative lesion extending from the lower gastric body to the antrum. (**C**) Upper gastrointestinal contrast study demonstrating an irregular lesion at the same site (yellow circle).

**Fig. 2:**
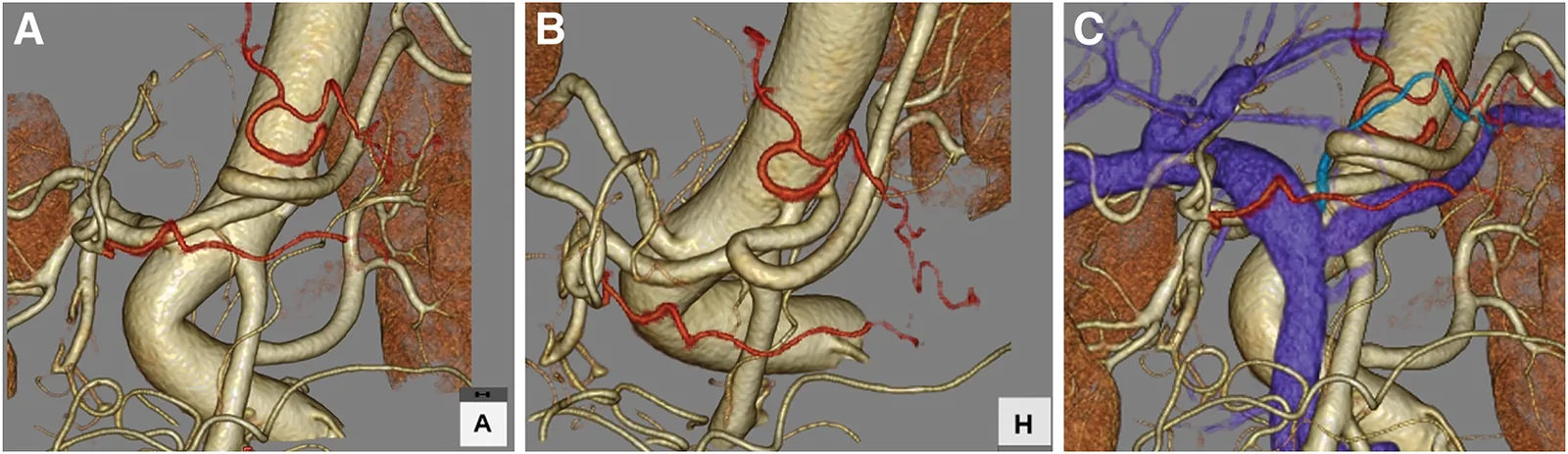
Preoperative 3D-CTA. (**A**, **B**) The left gastric, splenic, common hepatic, and superior mesenteric arteries arise independently from the abdominal aorta. (**C**) 3D reconstruction demonstrating the spatial relationship between the CHA and the portal vein, with the CHA coursing dorsal to the portal vein. CHA, common hepatic artery

A 77-year-old woman with no significant medical or family history and no history of smoking or alcohol use presented with persistent epigastric pain and was referred to our hospital. Physical examination revealed a flat and soft abdomen without tenderness, rebound pain, or peritoneal signs. Laboratory tests, including hematological and biochemical parameters, were within normal limits. Tumor marker levels were also within the reference ranges (CEA, 2.8 ng/mL; CA19-9, 12.9 U/mL). Upper gastrointestinal endoscopy revealed an irregular ulcerative lesion extending from the lower gastric body to the antrum (**Fig. 1**). Histopathological examination of the biopsy specimen revealed poorly differentiated adenocarcinoma with a signet-ring cell component. Contrast-enhanced CT showed localized gastric wall thickening corresponding to the primary lesion, with no evidence of distant metastasis, ascites, or clinically apparent lymph node enlargement. Abdominal contrast-enhanced CT revealed an atypical vascular anatomy, in which the LGA, SA, CHA, and SMA independently originated from the abdominal aorta. Furthermore, the CHA coursed dorsal to the portal vein (**Fig. 2**). Based on these findings, the patient was diagnosed with gastric cancer (cT3N0M0, cStage IIB, according to the eighth edition of the UICC TNM classification), and robotic distal gastrectomy with D2 lymphadenectomy was planned with careful consideration of the vascular anomaly.^8)^

### Surgical procedure

**Fig. 3:**
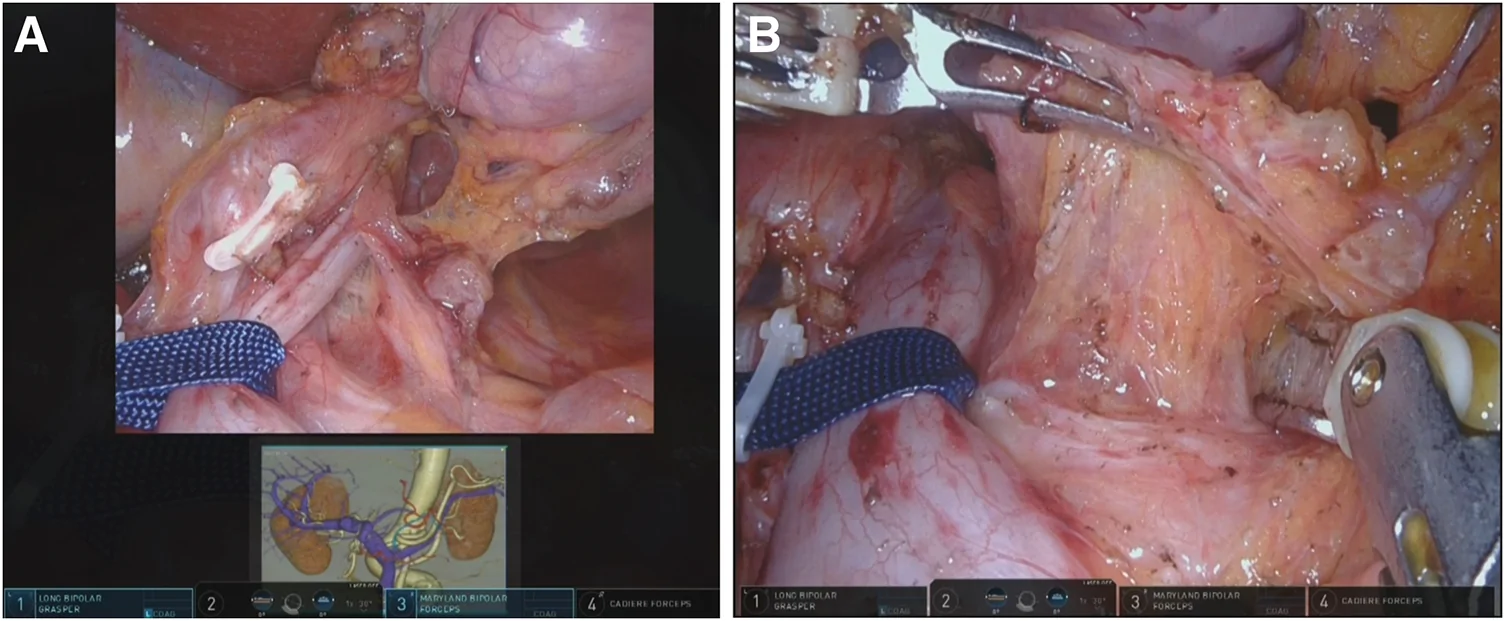
Intraoperative findings during robotic suprapancreatic lymph node dissection. (**A**) The portal vein is encircled with a tape to allow controlled retraction with reference to preoperative imaging. (**B**) Portal vein retraction secures an adequate operative field, enabling safe and precise dissection of the #8a lymph nodes along the CHA. CHA, common hepatic artery

Robotic distal gastrectomy with D2 lymph node dissection followed by Billroth II reconstruction was performed using the da Vinci Surgical System (Intuitive Surgical, Sunnyvale, CA, USA). Preoperative 3D CT images displayed on the surgeon console were used as intraoperative references for assessing the vascular anatomy and identifying the dissection planes. After duodenal transection, suprapancreatic lymph node dissection was initiated. Careful exploration revealed an inverted anatomical relationship, with the portal vein located ventral to the CHA. Under standard exposure, the portal vein limited the operative field, making adequate dissection plane establishment for lymph nodes #8a and #12a (located ventral to the CHA and within the hepatoduodenal ligament, respectively) difficult. To overcome this limitation, the portal vein was encircled with a tape and carefully retracted, clearly exposing the CHA, the proper hepatic artery, and the surrounding suprapancreatic structures, thereby allowing for safe and precise suprapancreatic (#8a and #9) and #12a lymph node dissection without vascular or pancreatic injury (**Fig. 3**). The operation was completed in 4.5 h with minimal blood loss, without intraoperative complications, and the patient was discharged uneventfully on POD 10. The key steps of the procedure are shown in **Supplementary Video 1**.

## DISCUSSION

**Fig. 4:**
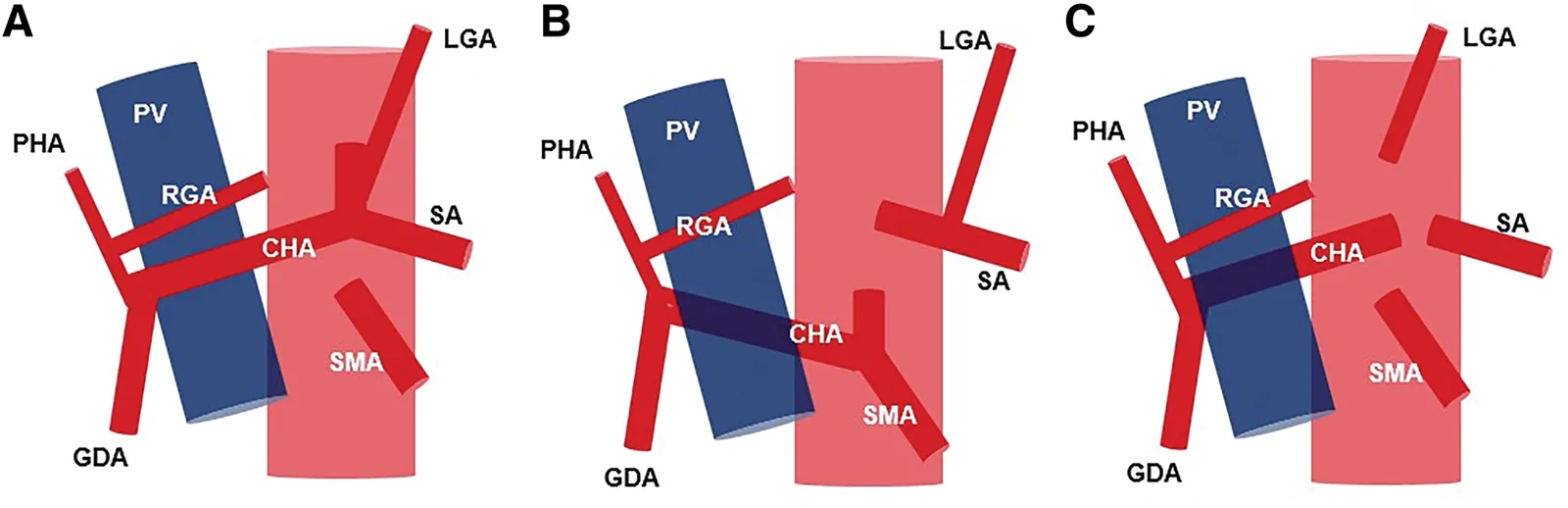
Schematic illustration of the vascular anatomy. (**A**) Normal anatomy showing the celiac trunk branching into the LGA, CHA, and SA, with the CHA coursing ventral to the PV. (**B**) Representative Adachi type VI pattern, in which the CHA arises from a hepatomesenteric trunk shared with the SMA. (**C**) The present case showing independent origins of the LGA, SA, CHA, and SMA from the abdominal aorta, with the CHA coursing dorsal to the PV. This configuration cannot be classified within existing systems. CHA, common hepatic artery; SMA, superior mesenteric artery; LGA, left gastric artery; PHA, proper hepatic artery; PV, portal vein; GDA, gastroduodenal artery; RGA, right gastric artery

We reported a case involving a rare vascular configuration in which the LGA, SA, CHA, and SMA arose independently from the abdominal aorta, and the CHA coursed dorsal to the portal vein. This combination of anomalous arterial origins and an unusual spatial relationship could not be adequately classified using existing systems. The Adachi classification primarily categorizes celiac arterial branching patterns, whereas the Michels classification focuses on hepatic arterial origin and does not incorporate the 3D relationship between the CHA and the portal vein.^4,5)^ In the present case, no celiac or hepatomesenteric trunk was formed; therefore, the anatomy only partially shared features with established categories^9)^ (**Fig. 4**).

Previous reports have shown that minimally invasive gastrectomy can be performed safely in patients with classifiable vascular anomalies, including Adachi type VI (group 26), when preoperative imaging clearly delineates the aberrant hepatic arterial anatomy.^10,11)^ However, operative strategies for anomalies that combine atypical arterial origins with unusual artery–vein relationships remain insufficiently described. In the present case, preoperative 3D-CTA demonstrated that the portal vein lay ventral to the CHA, allowing us to anticipate restricted access to the #8a and #12a lymph node regions.

Intraoperatively, portal vein taping enabled controlled retraction of the vein and exposed the ventral aspect of the CHA, thereby facilitating safe suprapancreatic lymph node dissection. Although portal vein taping is not routinely required, it may be useful when an anomalous vascular relationship obscures the conventional dissection plane. This strategy is particularly compatible with robotic surgery, in which stable retraction, magnified 3D visualization, and articulated instruments can support precise dissection in a confined operative field.

Celiac and superior mesenteric arterial variations have been reported in approximately 20%–40% of the general population.^9)^ At our institution, 234 patients underwent surgery for gastric cancer between January 2015 and August 2017, and preoperative 3D-CTA data were available for 201 patients. According to the Hiramatsu classification, among these 201 patients, 182 (90.5%) had a type IA major celiac arterial branching pattern, whereas 19 (9.5%) had other major branching patterns (**Supplementary Fig. S1**). However, the type IA category may still include additional arterial variations that are not represented by the major branching pattern. When such variations, including an aberrant left hepatic artery arising from the LGA, were considered, 130 patients (64.7%) had no arterial variation, whereas 71 (35.3%) had at least 1 arterial variation.

At our institution, a 3D-CTA image is routinely reconstructed from preoperative contrast-enhanced CT data in patients undergoing gastrectomy. Selective reconstruction based only on abnormalities suspected on a conventional axial CT image may overlook subtle arterial variations or unexpected 3D relationships between arteries and veins. Routine reconstruction can therefore provide clinically useful information for surgical planning without additional radiation exposure or contrast administration. Dedicated imaging, however, should be considered on an individual basis when suitable contrast-enhanced CT data are unavailable or contrast administration is contraindicated.

The CHA is an important landmark for suprapancreatic lymph node dissection, and unrecognized vascular variations may increase the risk of vascular or pancreatic injury and inadequate lymphadenectomy.^2,3,9)^ In the present case, the portal vein lay ventral to the CHA, obscuring the conventional dissection plane and limiting access to the #8a and #12a lymph nodes. Preoperative 3D-CTA findings enabled us to anticipate this anatomical difficulty, and portal vein taping provided adequate exposure for safe lymph node dissection. These findings emphasize the importance of tailoring the operative strategy to patient-specific vascular anatomy. This case therefore extends beyond the description of a rare vascular anomaly and demonstrates that detailed preoperative 3D-CTA assessment can facilitate individualized surgical planning and safe robotic gastrectomy, even when the vascular anatomy cannot be classified using existing systems.

## CONCLUSIONS

This case involved gastric cancer associated with a vascular anomaly that does not clearly fit within existing classification systems. However, safe robotic lymphadenectomy was achieved through detailed preoperative, 3D-CTA-based anatomical assessment and a planned surgical strategy incorporating portal vein taping. These findings highlight that, in anatomically complex situations, surgical safety depends on individualized preoperative planning and appropriate intraoperative strategies tailored to patient-specific anatomy.

## SUPPLEMENTARY MATERIALS

Supplementary Fig. S1 Distribution of celiac and superior mesenteric arterial branching patterns according to the Hiramatsu classification, based on preoperative 3D-CTA data from 201 patients with gastric cancer treated at our institution.

Supplementary Video 1 Intraoperative video showing exposure of the common hepatic artery by encircling and ventrally retracting the portal vein during robotic distal gastrectomy with D2 lymphadenectomy.

## DECLARATIONS

### Availability of data and materials

All data generated or analyzed during this study are included in this article.
